# CD160 serves as a negative regulator of NKT cells in acute hepatic injury

**DOI:** 10.1038/s41467-019-10320-y

**Published:** 2019-07-22

**Authors:** Tae-Jin Kim, Gayoung Park, Jeongmin Kim, Seon Ah Lim, Jiyoung Kim, Kyungtaek Im, Min Hwa Shin, Yang-Xin Fu, Maria-Luisa Del Rio, Jose-Ignacio Rodriguez-Barbosa, Cassian Yee, Kyung-Suk Suh, Seong-Jin Kim, Sang-Jun Ha, Kyung-Mi Lee

**Affiliations:** 1 Department of Biochemistry and Molecular Biology, Korea University College of Medicine, Seoul, 02841 Republic of Korea; 2 Department of Medicine, Section of Dermatology, The University of Chicago, Chicago, IL 60637 USA; 3 Department of Pathology, University of Texas Southwestern Medical Center, Dallas, TX 75390 USA; 4 Transplantation Immunobiology Section, Institute of Biomedicine, University of Leon, Leon, 24071 Spain; 5 Department of Melanoma Medical Oncology and department of Immunology, UT MD Anderson Cancer Center, Houston, TX 77054 USA; 6 Department of Surgery, Seoul National University College of Medicine, Seoul, 03080 Republic of Korea; 7 Precision Medicine Research Center, Advanced Institutes of Convergence Technology, Seoul National University, Suwon, Gyeonggi-do 16229 Republic of Korea; 8 Department of Biochemistry, College of Life Science and Biotechnology, Yonsei University, Seoul, 03722 Republic of Korea; 9 Department of Biomedical Engineering, Center for Bio-Integrated Electronics, Simpson Querrey Institute for BioNanotechnology, Northwestern University, Evanston, IL 60208 USA

**Keywords:** Acute inflammation, NKT cells, Signal transduction, Liver diseases

## Abstract

CD160 and BTLA both bind to herpes virus entry mediator. Although a negative regulatory function of BTLA in natural killer T (NKT) cell activation has been reported, whether CD160 is also involved is unclear. By analyzing CD160^−/−^ mice and mixed bone marrow chimeras, we show that CD160 is not essential for NKT cell development. However, CD160^−/−^ mice exhibit severe liver injury after in vivo challenge with α-galactosylceramide (α-GalCer). Moreover, CD160^−/−^ mice are more susceptible to Concanavalin A challenge, and display elevated serum AST and ALT levels, hyperactivation of NKT cells, and enhanced IFN-γ, TNF, and IL-4 production. Lastly, inhibition of BTLA by anti-BTLA mAb aggravates α-GalCer-induced hepatic injury in CD160^−/−^ mice, suggesting that both CD160 and BTLA serve as non-overlapping negative regulators of NKT cells. Our data thus implicate CD160 as a co-inhibitory receptor that delivers antigen-dependent signals in NKT cells to dampen cytokine production during early innate immune activation.

## Abstract (web summary)

BTLA is established as a negative regulator of natural killer T (NKT) cell function, and share its ligand HVEM with CD160. Here the authors show, by analyzing NKT activation in CD160-deficient mice or with BTLA blockade, that CD160 synergizes with BTLA to negatively regulate NKT cells during hepatic injury.

## Introduction

CD160 is a glycosylphosphatidylinositol-anchored Ig domain protein that is expressed on almost all intestinal intraepithelial lymphocytes (IELs), γδ T (gamma delta T) cells, NK (natural killer) cells, and a minor subset of CD4^+^ and CD8^+^ T cells^1–3^. CD160 has been shown to bind MHC Class I at low affinity and herpes virus entry mediator (HVEM) at high affinity, and shares its ligand HVEM with BTLA in both T cells and NK cells^4,5^. While activation of HVEM signaling by LIGHT, BTLA, or CD160 binding enhances Ag-induced T cell proliferation and cytokine production^6–8^, engagement of BTLA/CD160 by HVEM provides negative signals to T cells via BTLA/CD160^4,5^. Furthermore, CD160 has been reported to be a marker for T cell exhaustion, and increased CD160 expression in CD8^+^ T cells is associated with T cell exhaustion in chronic viral infection models^9–11^. In contrast, CD160 cross-linking by MHC ligands (HLA-C) co-stimulates CD8^+^ T cells and activates NK cell cytotoxicity and cytokine production^12^. Therefore, CD160 can function as both a co-activating and co-inhibitory receptor, depending on which receptor/ligand is operating in the context of neighboring interactions.

Innate-like NKT cells express the NK1.1 marker as well as other typical NK receptors. Upon stimulation through their TCR, they rapidly produce substantial amounts of cytokines. Unlike NK cells, NKT cells develop in the thymus and express a rearranged TCR, whereas most of the population expresses canonical Vα14–Jα18 TCRα chains associated with Vβ8, Vβ7 or Vβ2^13–15^. The best-characterized population is a NKT set composed of αβ T cells specific for microbial lipids presented by the MHC class I-like CD1d molecule, which is expressed on antigen-presenting cells (APCs) including dendritic cells (DCs) and Kupffer cells (KC)^16^. After stimulation with a CD1d-bound antigen, NKT cells produce large amounts of pro- and anti-inflammatory cytokines within hours^17,18^. Interleukin (IL)-2, interferon (IFN)-γ, and tumor necrosis factor (TNF)-α lead to a pro-inflammatory T helper 1 (Th1) response, while cytokines such as IL-4, IL-5, IL-6, IL-10, and IL-13 promote an immunoregulatory T helper 2 (Th2) response. Increasing evidence suggests that NKT cells are essential for microbial defense and the initiation of adaptive immune responses in regulating autoimmune responses.

Activation of NKT cells is triggered by ligation of the semi-invariant TCR along with an array of co-stimulatory (CD28, CD40L, CD70, OX40, and 41BB) and co-inhibitory receptors (CTLA-4, PD-L1, GITR, and BTLA). Although it has been established in vitro and in vivo that BTLA is expressed on NKT cells and functions as a negative regulator in early innate immune responses, the role of CD160 in NKT cells is not fully understood due to the lack of proper blocking reagents and gene-deficient animal models.

Using CD160^−/−^ mice and a mixed bone marrow chimera generated in a lethally irradiated WT host, we evaluate the role of CD160 in BTLA/HVEM interactions. Here, we present data showing that the CD160/HVEM signaling axis delivers a BTLA-independent negative signal to NKT cells in both α-GalCer and Con A-induced acute hepatitis.

## Results

### α-GalCer stimulation causes upregulation of CD160 in vivo

**Fig. 1:**
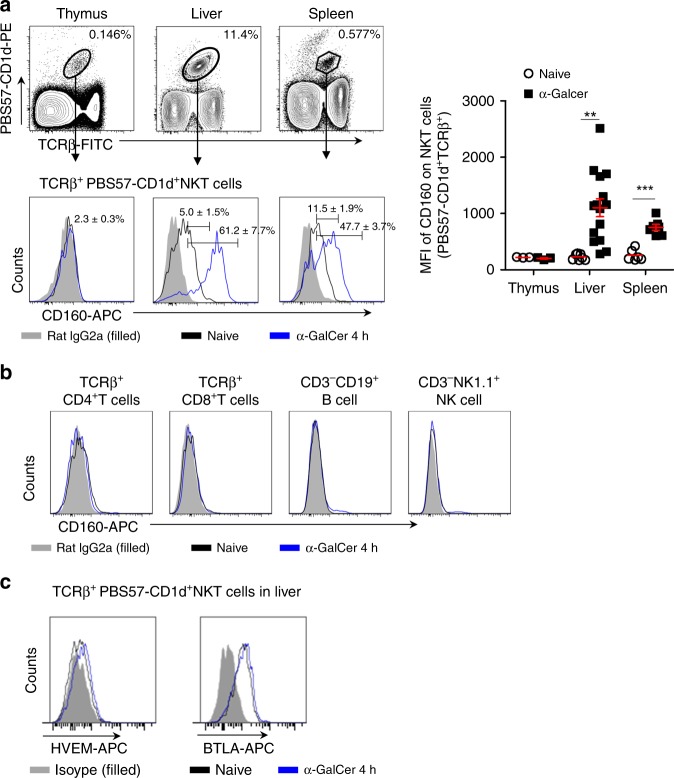
Upregulation of CD160 on NKT cells after α-GalCer stimulation in vivo. **a**, **b** CD160 expression on NKT cells from the thymus, liver, and spleen 4 h before and after α-GalCer challenge in vivo. Lymphocytes were stained with anti-TCR-β, PBS57-CD1d tetramer, and anti-CD160 APC mAb or control IgG. **a** Representative flow cytometry plots of CD160 expression and summary of the frequency of CD160 positivity in gated TCRβ^+^ PBS57-CD1d tetramer^+^ from thymus, liver, and spleen NKT cells for 4 h before and after i.p. injection with α-GalCer (2 μg/mice). The graph represents the mean florescence intensity of CD160 expression of NKT cells from the thymus, liver, and spleen (*n* = 3–15 per group). **b** Flow cytometry plots of CD160 expression in TCRβ^+^CD4^+^, TCRβ^+^CD8^+^, CD3^−^CD19^+^, and CD3^−^NK1.1^+^ cells of liver MNCs 4 h before and after α-GalCer injection. Results are representative of three independent experiments. **c** Representative flow cytometry plots of HVEM or BTLA expression in gated TCRβ^+^ PBS57-CD1d tetramer^+^ NKT cells from liver MNCs. All data are presented as mean ± S.E.M. *n* = 3. *P < 0.05, **P < 0.01, ***P < 0.001 with an unpaired two-tailed *T*-test

We first analyzed the surface expression of CD160 on NKT cells isolated from the thymus, liver, and spleen of C57BL/6 mice by flow cytometry. NKT cells were gated according to previous reports^19^ and the expression of CD160 on CD45^+^ TCRβ^+^ PBS57-CD1d-Tetramer^+^ NKT cells was analyzed in the primary and secondary lymphoid organs (Fig. 1a). NKT cells expressing surface CD160 comprised of 2.3 ± 0.3% of cells in the thymus, 5.0 ± 1.5% in the liver, and 11.5 ± 1.9% in the spleen at naive status (Fig. 1a). In an in vivo challenge of C57BL/6 mice with α-GalCer, the percentage of CD160^+^ NKT cells increased from 5.0 ± 1.5% to 61.2 ± 7.7% in the liver and from 11.51 ± 1.9 to 47.7 ± 3.7% in the spleen within 4 h, with significantly increased MFI (Fig. 1a). In contrast, CD4^+ ^T, CD8^+^ T, B, and NK cells did not exhibit altered surface CD160 in this time frame (Fig. 1b). Interestingly, there was no specific upregulation in other co-stimulatory or co-inhibitory receptors on NKT cells, including BTLA, HVEM, 41BB, PD-1, OX40, CD28, CTLA-4, and CD95 (Fig. 1c and Supplementary Fig. 1). These findings indicate that NKT cells specifically upregulate CD160 expression during α-GalCer-induced hepatitis, accompanied by their early inflammatory processes.

### CD160 does not affect the development status of murine NKT cells

**Fig. 2:**
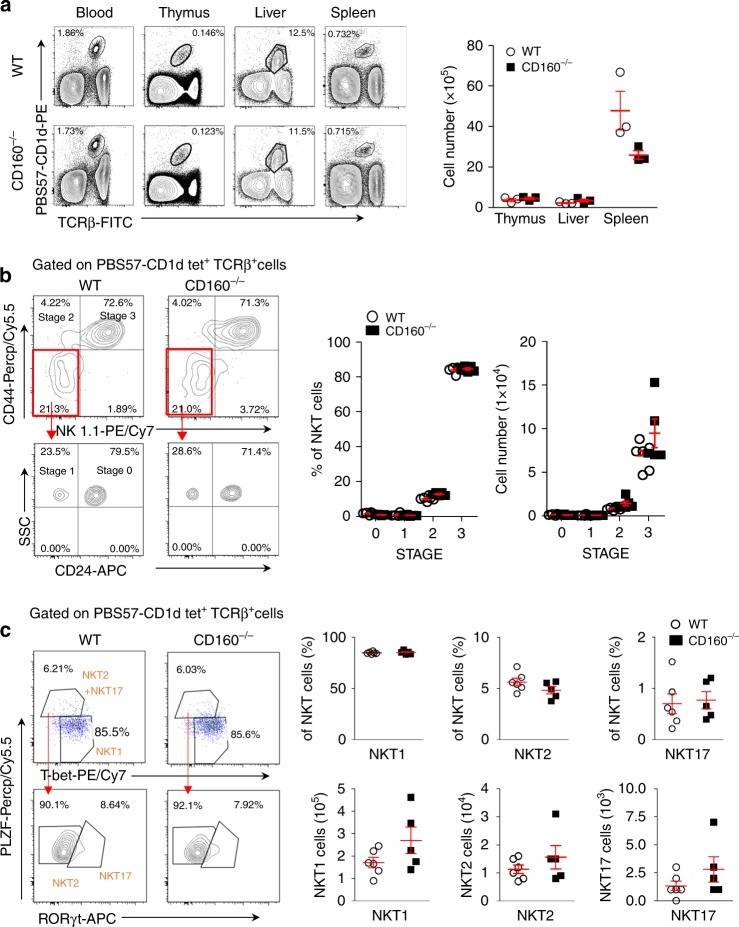
Absence of CD160 does not affect the development of NKT cells. **a** Lymphocytes from blood, thymus, liver, and spleen in WT and CD160^−/−^ mice were stained with anti-TCRβ FITC and PBS57-CD1d tetramer PE and analyzed by flow cytometry. Results are representative of three independent experiments. The absolute number of TCRβ^+^ PBS57-CD1d Tetramer^+^ NKT cells in the thymus, liver, and spleen was calculated based on the percentage of each population shown in (*n* = 3 per group). **b** Flow cytometry analysis of CD44 and NK1.1 expression among total NKT cells from the thymus of WT and CD160^−/−^ mice. Results are representative of three independent experiments. The percentages and numbers of thymic NKT cells at stage 0 (NK1.1^−^, CD44^−^, CD24^+^), stage 1 (NK1.1^−^, CD44^−^, CD24^−^), stage 2 (NK1.1^−^, CD44^+^), and stage 3 (NK1.1^+^, CD44^+^), based on flow cytometry analysis. Results are representative of three independent experiments. (*n* = 5–6 per group). **c** Thymic NKT cells were gated on PLZF, T-bet and RORγt and the percentage and absolute numbers of NKT1, NKT2, and NKT17 were calculated (*n* = 5–6 per group). All data are presented as mean ± S.E.M. *P < 0.05, **P < 0.01, ***P < 0.001 with an unpaired two-tailed *T*-test

To determine the role of CD160 in the development of NKT cells, we examined the frequency and number of NKT cells in the blood, thymus, liver, and spleen in CD160^−/−^ mice. When compared to WT mice, CD160^−/−^ mice did not show any significant difference in the proportion of NKT cells in each organ tested (Fig. 2a). Furthermore, no changes in the developmental stages of NKT cells, defined by stage 0 (CD24^+^CD44^−^NK1.1^−^), stage 1 (CD44^low^, NK1.1^−^), stage 2 (CD44^+^, NK1.1^−^), and stage 3 (CD44^+^, NK1.1^+^)^20^, were noted as compared to WT mice (Fig. 2b).

The transcription factor pro-myelocytic leukemia zinc finger (PLZF) is an early transcriptional regulator of NKT cell development^21^. We further analyzed NKT cell subsets using a combination of lineage-specific transcription factors, as described previously^22^. The combination of PLZF, T-bet, and RORγt separated NKT cells into three subsets that produced distinct cytokines^22^. Using intracellular staining with Abs against PLZF, T-bet and RORγt, we defined NKT1 (T-bet^+^ PLZF^low^ RORγt^−^), NKT2 (T-bet^−^ PLZF^+^ RORγt^−^), and NKT17 (T-bet^−^ PLZF^int^ RORγt^+^) cells by flow cytometry. The frequency and the absolute number of NKT1, NKT2, and NKT17 cells did not differ significantly between WT and CD160^-/-^ mice in the thymus or in the liver and spleen (Fig. 2c and Supplementary Fig. 2). Collectively, these findings indicate that CD160 is not essential for the development and differentiation of NKT cells.

### CD160^−/−^ mice exhibit severe α-GalCer-induced liver injury

**Fig. 3:**
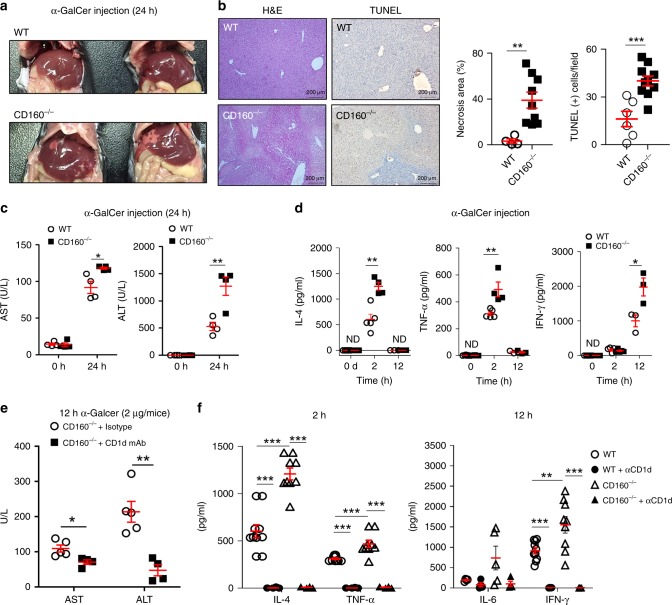
CD160^−/−^ mice are more susceptible to α-GalCer-induced liver injury. **a** Representative images of livers of WT and CD160^−/−^ mice 24 h following i.p. injection of α-GalCer (2 μg). **b** H&E and TUNEL staining of liver sections in WT and CD160^−/−^ mice. The percentage necrotic area (*n* = 4–9 per group) and TUNEL (+) cells/field are shown as mean ± S.E.M. (Random area, *n* = 6–10 per group). Scale bars: 200 μm. **c** Serum transaminase activity (AST, ALT) 0 and 24 h following i.p. injection of α-GalCer (2 μg). Data are presented as mean ± S.E.M. (*n* = 4 per group). **d** Serum was collected from WT and CD160^−/−^ mice at different time points following 2 μg of α-GalCer injection and cytokines measured by CBA (ND, not detected). Results are representative of three independent experiments (*n* = 3–8 per group). **e**-**f** Mice were injected with anti-CD1d mAb (20H2) 24 h before α-GalCer injection. **e** Analysis of serum transaminase activities (AST, ALT) 12 h following i.p. injection of α-GalCer (2 μg) with anti-CD1d or isotype control administration. Data are presented as mean ± S.E.M. (*n* = 4–5 per group). **f** Analysis of serum levels of IL-4, TNF-α, IL-6, and IFN-γ in WT and CD160^−/−^ mice at 2 h and 12 h after i.p. injection of α-GalCer (2 μg). The levels of various cytokines in serum were measured via CBA analysis (*n* = 4–10 per group). All data are presented as mean ± S.E.M. *P < 0.05, **P < 0.01, ***P < 0.001 with an unpaired two-tailed *T*-test

To test the role of CD160 in the regulation of NKT functions, we adopted α-GalCer-induced hepatitis mouse model, which resembles human autoimmune acute hepatitis^23^. Induction of hepatitis was evaluated in the liver tissues of WT and CD160^−/−^ mice by liver morphology study, H&E staining and TUNEL assay at 24 h following α-GalCer injection (Fig. 3a, b). CD160^−/−^ mice demonstrated massive hepatic necrosis with elevated AST and ALT levels in serum (Fig. 3a–c) compared with WT mice. The serum levels of NKT-dependent cytokines IL-4, TNF-α, and IFN-γ were significantly higher in CD160^−/−^ mice than in WT mice 2 h or 12 h following an α-GalCer challenge (Fig. 3d). Increased tissue injury and elevated serum cytokines in CD160^−/−^ mice were not found to be associated with elevated infiltration of CD11C^+^MHCII^+^ DCs, Ly6G^+^CD11b^+^ neutrophils, Ly6C^high^ CD11b^+^ monocytes, or F4/80^+^CD11b^+^ Ly6G^−^ Kupffer cells in the liver, as the percentages of these innate immune cells were comparable in CD160^−/−^ mice and WT mice (Supplementary Fig. 3). Furthermore, the surface levels of CD160 ligand, HVEM (Supplementary Fig. 4), and CD40, CD1d, PD-L1, and CD80 (Supplementary Fig. 5) in liver MNCs were comparable in α-GalCer-administered WT and CD160^−/−^ mice (Supplementary Fig. 5), suggesting that hyperactivation of NKT cells in CD160 mice was not likely due to changes in the expression of co-stimulatory or co-inhibitory receptors. These data indicate that CD160 normally acts in the early immune response by dampening cytokine production, thereby protecting against α-GalCer-induced liver injury.

We next investigated whether CD160 regulates antigen-dependent signals downstream of α-GalCer presented on CD1d, not from other cell surface activating receptors. Therefore, we injected anti-CD1d mAbs 24 h prior to α-GalCer injection to block CD1d-mediated signaling, and AST/ALT and related cytokine (IL-4, TNF-α, IL-6, and IFN-γ) levels in the blood were measured in WT and CD160^−/−^ mice. As shown in Fig. 3e, f, anti-CD1d mAb-treated CD160^−/−^ mice demonstrated significantly decreased liver damage, showing reduced AST/ALT levels (Fig. 3e). Moreover, blocking CD1d abrogated α-GalCer-induced cytokine secretion in both WT and CD160^−/−^ mice (Fig. 3f), suggesting that CD160 specifically regulates α-GalCer-induced NKT signals presented on CD1d.

### Uncontrolled activation of the NKT cells in CD160^−/−^ mice

**Fig. 4:**
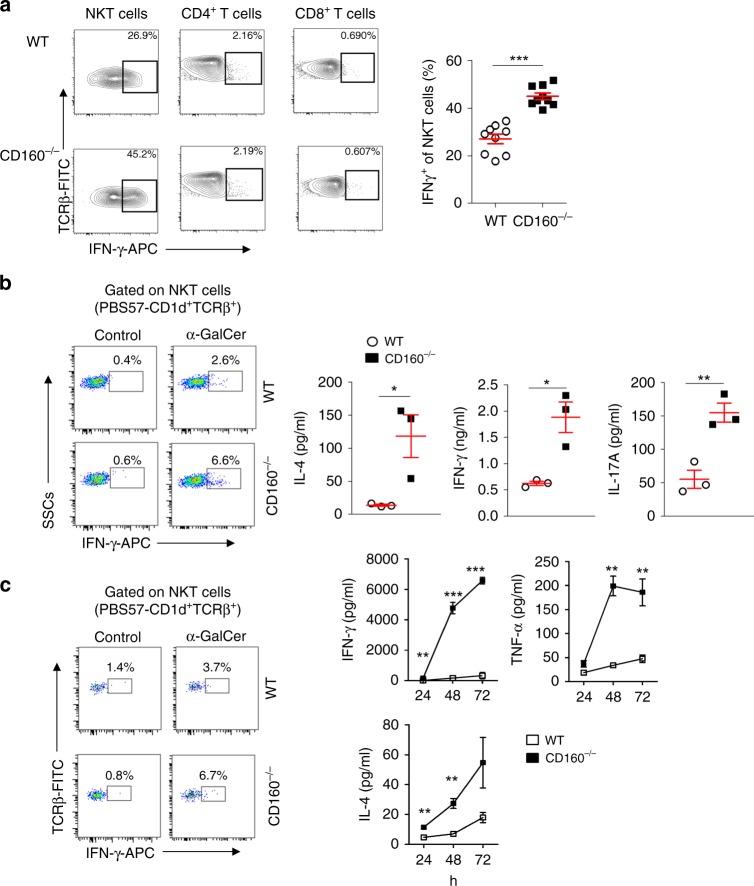
Expression of IFN-γ and surface co-receptors on NKT cells. **a** Representative flow cytometric analyses of intracellular IFN-γ expression in liver NKT, CD4^+^, and CD8^+^ T cells isolated from WT and CD160^−/−^ mice injected with 2 μg of α-GalCer for 4 h. Liver MNCs were treated with Brefeldin A to block cytokine release for the last 6 h in culture for intracellular IFN-γ staining. The graphs represent the frequency of IFN-γ-expressing NKT cells (*n* = 9 per each group). **b** Liver MNCs (2 × 10^5^) from littermates of WT and CD160^−/−^ mice were cultured with α-GalCer (100 ng/ml) for 24 h. Cells were treated with Brefeldin A to block cytokine release for the last 6 h in culture for intracellular IFN-γ staining. Levels of IL-4, IFN-γ, and IL-17A in the culture supernatants at 24 h were measured by CBA. Results are representative of three independent experiments (*n* = 3 per group). **c** Splenocytes (3 × 10^5^ cells) from littermates of WT and CD160^−/−^ mice were cultured with α-GalCer (100 ng/ml) and IL-2 (50 U / ml) for 24 h. Cells were fixed and permeabilized prior to intracellular staining. Levels of IL-4, IL-6, IFN-γ, and TNF-α in the culture supernatants for 24, 48, and 72 h were measured by CBA (*n* = 3 per group). All data are presented as mean ± S.E.M. *P < 0.05, **P < 0.01, ***P < 0.001 with an unpaired two-tailed *T*-test

To establish that the cytokines produced in CD160^−/−^ mice by α-GalCer were not coming from activated CD4^+^ and CD8^+ ^T cells^24^, we injected mice with α-GalCer and isolated liver MNCs after 4 h for intracellular detection of IFN-γ. As seen in Fig. 4a, IFN-γ expression was significantly increased on NKT cells, not CD4^+^ and CD8^+^ T cells, in response to α-GalCer (Fig. 4a). These results indicate that increased IFN-γ in CD160^−/−^ mice during α-GalCer-induced hepatitis was primarily due to activation of NKT cells, not CD4^+^ or CD8^+^ T cells.

We recapitulated an acute α-GalCer-induced hepatic inflammation model in an in vitro setup with isolated liver MNCs to confirm the role of CD160. Liver MNCs were isolated from WT and CD160^−/−^ mice and stimulated with α-GalCer for 24 h in vitro. NKT cells from CD160^−/−^ mice secreted higher levels of IL-4, IFN-γ, and IL-17A than those of WT mice, consistent with our in vivo data (Fig. 4b). Similar results were obtained with splenocytes harvested from WT and CD160^−/−^ mice, showing a time-dependent increase in IL-4, IFN-γ, and TNF-α up to 72 h (Fig. 4c). These findings suggest that CD160 receptors on NKT cells are crucial for regulating the production of inflammatory cytokines upon α-GalCer stimulation.

### CD160 and BTLA are non-overlapping co-inhibitory receptors

**Fig. 5:**
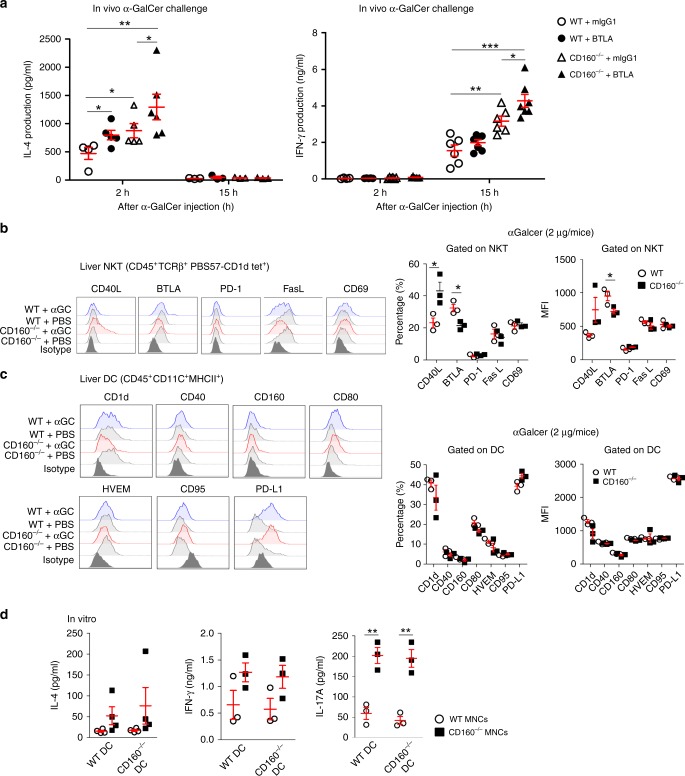
α-GalCer challenge in the presence of BTLA blocking mAbs in CD160^−/−^ mice. **a** mIgG1 or anti-BTLA mAbs were injected into WT or CD160^−/−^ mice 2 h prior to α-GalCer in vivo challenge and serum levels of IL-4 and IFN-γ were measured by CBA 2 and 15 h post α-GalCer challenge (*n* = 3–7 per group). Surface expression of co-stimulatory and co-inhibitory receptors on NKT (**b**) and dendritic cells (**c**) from WT and CD160^−/−^ mice 4 h before and after 2 μg of α-GalCer challenge in vivo. Left: Representative FACs plots from three independent experiments. Right: Average percentages or MFIs were plotted for each receptor as bar graphs (*n* = 3–5 per each group). **d** Co-cultures of WT or CD160^−/−^ liver MNCs with WT or CD160^−/−^ BMDCs for 48 h in vitro. BMDCs were pulsed with α-GalCer for 16 h prior to co-culture (*n* = 3–4 per group). All data are presented as mean ± S.E.M. *P < 0.05, **P < 0.01, ***P < 0.001 with an unpaired two-tailed *T*-test

Since BTLA, which is constitutively expressed on NKT cells, also exerts its inhibitory function through HVEM, we next examined whether CD160^−/−^ mice show additional elevated cytokine secretion in the presence of BTLA/HVEM interaction. To test this, we injected blocking anti-BTLA mAbs (clone: pj196) into WT and CD160^−/−^ mice prior to α-GalCer challenge in vivo (Fig. 5a). While the injection of anti-BTLA blocking mAbs in WT mice leads to a subtle increase in the level of IL-4 and IFN-γ, BTLA blocking in CD160^−/−^ mice caused additional increases in IL-4 and IFN-γ at 2 h and 15 h post α-GalCer injection, respectively. These data suggest that both CD160 and BTLA play a non-redundant co-inhibitory role in mice regulating liver inflammation during α-GalCer-induced acute hepatitis.

We next determined whether accelerated production of cytokines from CD160^−/−^ NKT cells in the liver was associated with upregulation of co-stimulatory or downregulation of co-inhibitory receptors on NKT cells^25–31^. When analyzed ex vivo, surface expression of CD154 (CD40L) was found to be significantly increased while that of BTLA was decreased on CD160^−/−^ NKT cells upon α-GalCer challenge. No obvious differences in PD-1, FasL, or CD69 expression were observed between WT and CD160^−/−^ NKT cells (Fig. 5b). These data suggest that upregulation of a CD40L/CD40 co-stimulatory pathway in NKT cells might have contributed to enhanced pro-inflammatory responses in the absence of CD160. Uncontrolled activation of NKT cells might have been influenced by defective DCs in CD160^−/−^ mice. To assess whether the activation status of DCs was altered in the absence of CD160, we measured the levels of multiple ligands on DCs, including CD1d, CD40, CD80, CD95, PD-L1, and HVEM, during α-GalCer-induced hepatitis. As seen in Fig. 5c, no significant changes in surface expression were noted between WT and CD160^−/−^ DCs. To further assess whether α-GalCer-induced NKT cell activation was dependent on DCs, liver MNCs from WT and CD160^−/−^ mice were co-cultured with α-GalCer-pulsed DCs in vitro for 48 h and production of cytokines was measured by CBA analysis. As shown in Fig. 5d, CD160^−/−^ liver MNCs produced high levels of IL-4, IFN-γ, and IL-17A regardless of whether WT or CD160^−/−^ DCs were present (Fig. 5d). Therefore, aberrant activation of NKT cells, but not surrounding APCs, was primarily responsible for acute inflammation during α-GalCer-induced hepatitis in the absence of CD160.

### Uncontrolled NKT activation was due to intrinsic defects

**Fig. 6:**
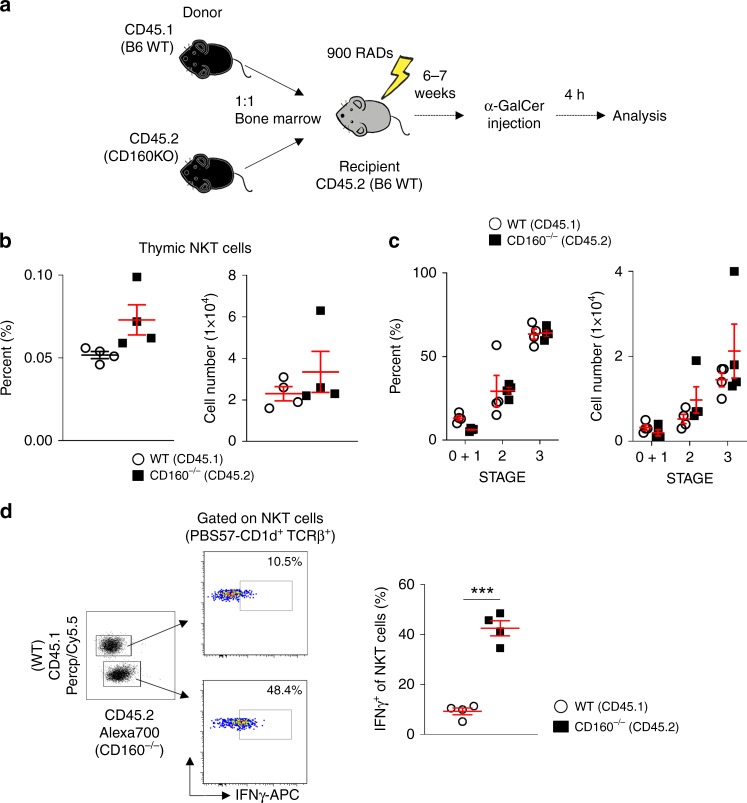
CD160^−/−^ NKT cells show intrinsic defects during acute hepatitis. **a** Mixed bone marrow (BM) chimera mice were generated using a 1:1 ratio of WT (CD45.1) and CD160^−/−^(CD45.2) BM cells for transfer into WT hosts; 6–7 weeks later, mice were injected with 2 μg of α-GalCer for 4 h. **b**-**c** Thymic NKT cells were analyzed by multiparametric flow cytometry using TCRβ, CD1d-tetramer, CD44, NK1.1, CD45.1, and CD45.2 to identify NKT cells in mixed BM chimera mice (*n* = 4 per each group). **d** Representative FACS plots of IFN-γ in NKT cells following in vivo challenge with 2 μg of α-GalCer. All data represent the mean ± S.E.M. Results are representative of two independent experiments (*n* = 4 per group). *P < 0.05, **P < 0.01, ***P < 0.001 with a paired *T*-test

To investigate whether the phenotypic defects seen in CD160^−/−^ mice upon α-GalCer challenge is intrinsic to NKT cells, we generated competitive mixed bone marrow chimera mice. For this, the bone marrow of CD45.2^+^CD160^−/−^ mice and “competitor” bone marrow from CD45.1^+^ WT mice were mixed at a 1:1 ratio and injected into γ-irradiated WT C57BL/6 mice (Fig. 6a). The reconstituted mice were then sacrificed six to seven weeks post-injection and analyzed. The developing NKT cell compartment of the chimera mice was normal, as seen by the expression of CD44, NK1.1, and CD24 on thymocytes (Fig. 6b, c). Upon α-GalCer injection of mixed chimeric mice, secretion of IFN-γ was greatly enhanced in CD45.2^+^ CD160^−/−^ NKT cells compared with CD45.1^+^ WT NKT cells (Fig. 6d). These data indicate that the cytokine defects are intrinsic to NKT cells, and not due to extrinsic defects.

### CD160^−/−^ mice suffer from Concanavalin A-induced hepatitis

**Fig. 7:**
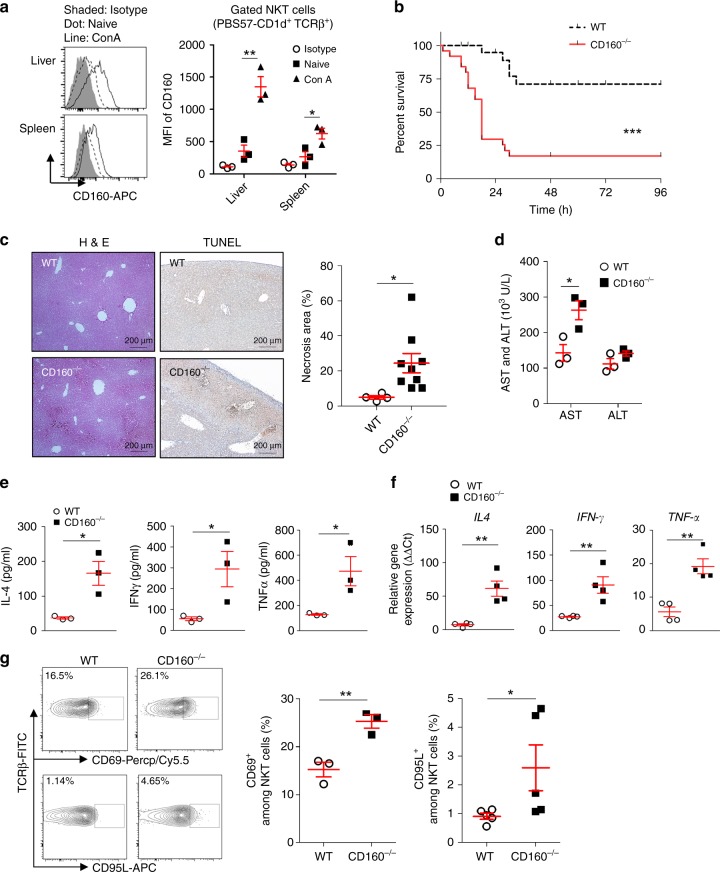
Susceptibility to Con A-induced hepatitis in CD160^−/−^ mice. **a** Representative histograms showing CD160 expression in gated PBS57-CD1d tetramer^+^ TCRβ^+^ NKT cells from the liver and spleen 4 h before and after i.v. injection with Con A (15 mg/kg). The graph represents average mean fluorescence intensities (MFI) of CD160 expression of NKT cells from liver and spleen (*n* = 3 per group). **b** WT and CD160^−/−^ mice were intravenously injected with Con A (30 mg/kg), and the rate of survival was recorded (*n* = 21–25 per group). *P < 0.05, **P < 0.01, ***P < 0.001 with a Log-rank (Mantel–Cox) Test. **c** The percentage of necrotic area and TUNEL (+) cells/field are shown as mean ± S.E.M. (*n* = 4–9 per group). Scale bars: 200 μm. **d** WT and CD160^−/−^ mice were intravenously injected with Con A (15 mg/kg), and serum was obtained to measure AST and ALT levels at 2 h (*n* = 3 per group). **e** Analysis of serum levels of IL-4, IFN-γ, and TNF-α in WT and CD160^−/−^ mice 4 h after intravenous injection of Con A (15 mg/kg) by CBA and data presented as the mean ± S.E.M. (*n* = 3 per group). **f** Quantitative RT-PCR analysis for *IL-4, IFN-γ*, and *TNF-α* mRNA levels in the livers of WT and CD160^−/−^ mice 4 h after intravenous injection of Con A (15 mg/kg). Gene expression was normalized to *HPRT* mRNA levels in each sample (*n* = 4 per group). **g** Representative flow cytometry analysis showing expression of CD69 and CD95 Ligand in NKT cells from WT and CD160^−/−^ mice 4 h after Con A treatment (15 mg/kg). Graphs represent the percentage of the CD69 and CD95L positive portions in NKT cells among liver MNCs from WT and CD160^−/−^ mice (*n* = 3–5 per group). All data are representative of three different experiments and presented as mean ± S.E.M. *P < 0.05, **P < 0.01 with an unpaired two-tailed *T*-test or Mann–Whitney test

We next investigated whether CD160 contributes to NKT cell activation in more physiologic settings of acute hepatitis. Although α-GalCer presented by CD1d provides specific NKT-dependent activation both in vivo and in vitro, Con A-induced murine hepatitis is a well-documented experimental model for human autoimmune acute hepatitis or viral hepatitis^32,33^. To define the role of CD160 on NKT cells in an autoimmune hepatitis model, we first measured surface CD160 expression on NKT cells after Con A (15 mg/kg) administration in vivo. Similar to α-GalCer stimulation, Con A administration resulted in elevation of surface CD160, up to 3-fold, on PBS57-CD1d^+^ TCRβ^+^ NKT cells isolated from the liver and spleen in WT mice (Fig. 7a). To further investigate the role of CD160 receptor in an autoimmune hepatitis model, we injected a lethal dose (30 mg/kg) of Con A i.v. into WT and CD160^−/−^ mice with and followed their survival over time. As seen in Fig. 7b, upon Con A challenge, over 75% of CD160^−/−^ mice died within 24 h while the majority of WT mice survived (Fig. 7b). As seen in the α-GalCer challenge, CD160^−/−^ mice demonstrated aggravated tissue damage during Con A-induced hepatitis compared with WT mice, along with elevated AST and ALT in serum (Figs 7c, d). In addition, IL-4, IFN-γ, and TNF-α levels in CD160^−/−^ mice were significantly higher than in WT mice (Fig. 7e). The mRNA transcripts of *IL-4*, *IFN-γ*, and *TNF-α* were also significantly higher after Con A injection in CD160^−/−^ mice than WT, indicating that CD160 negatively regulates cytokine expression in NKT cells (Fig. 7f).

To show that this defect was coming from NKT cells, but not CD8^+ ^T cells, we performed in vivo depletion of CD8^+ ^T cells by injecting anti-CD8 mAbs 24 h before Con A injection. Regardless of CD8^+ ^T cell depletion, CD160^−/−^ mice showed a decreased rate of survival compared with WT mice (Supplementary Fig. 6). When we analyzed the activation status of hepatic NKT cells upon Con A stimulation, the early activation marker CD69 was increased significantly in the NKT cells of CD160^−/−^ mice (Fig. 7g). Upon Con A treatment, CD95 ligand expression on hepatic NKT cells was upregulated and hepatic injury was induced by CD95–CD95 ligand (L) cytotoxicity^34^. We found that CD160^−/−^ mice had higher CD95L on hepatic NKT cells than WT mice (Fig. 7g). Our results indicate that loss of CD160 leads to hyperactivation of NKT cells and liver inflammation via upregulation of CD95L. These results provide strong evidence that CD160 plays a critical role as a negative regulator in the activation of NKT cells.

## Discussion

Co-inhibition of antigen-induced signals is critical for the contraction of adaptive immune responses as well as for managing uncontrolled immune responses. Here, we report that CD160 serves as an important co-inhibitory receptor of NKT cells during early innate immune responses, preventing aberrant activation and subsequent tissue damage. While the absence of CD160 did not affect the normal development and differentiation processes of NKT cells in CD160^−/−^ mice, it shifted the balance of NKT cells toward more uncontrolled hyperactivation in the periphery and secondary lymphoid organs. In both α-GalCer- and Con A-induced murine hepatitis models, CD160^−/−^ mice suffered from severe inflammation with elevated IL-4, IL-6, IFN-γ, and TNF-α and could not fully recover from lethal Con A challenge. The inhibitory role of CD160 in NKT cells appears to be independent from BTLA, as simultaneous blocking of BTLA using specific anti-BTLA mAbs further elevated NKT-dependent cytokine production. These data show that both CD160 and BTLA serve as non-overlapping negative regulators of NKT cells during the early innate immune response.

The negative regulatory role of CD160 in NKT cells seen here is somewhat contradictory to its known co-stimulatory function in NK cells and CD8^+^ T cells^12,35,36^. However, it coincides with findings in CD4^+^ T cells, which have shown that in vitro cross-linking of CD160^37^ or its engagement by HVEM on APCs co-inhibited T cell activation^5,38–40^. Since NKT cells constitutively express BTLA as a strong inhibitory receptor transmitting signals from HVEM^26,41^, having CD160 as an additional negative receptor binding to HVEM appears quite redundant. However, one can speculate that NKT cells may utilize BTLA to bind HVEM during the initial phase of immune response, and then allow CD160 upregulation to strengthen binding to HVEM in the later phase. Although this hypothesis awaits experimental confirmation, our results shown here support the fact that CD160 acts as a critical co-inhibitory receptor in NKT cells, which is separate from the role of BTLA.

Since NKT cells rarely express HVEM on their surface (Fig. 1c), both antigenic signals and HVEM signals would have been delivered *in trans* from neighboring APCs, including DCs and Kupffer cells. HVEM also binds LIGHT, and HVEM/LIGHT interactions have been shown to co-stimulate T cell activation^42^. Engagement of HVEM on T cells by LIGHT expressed on DC co-stimulates CD8^+^ T cells and also induces proliferation and differentiation of CD4^+^ T cells. The HVEM/BTLA pathway, however, can downmodulate TCR-mediated signaling similarly in both T cell subsets. However, we found that NKT cells do not express a significant level of LIGHT on their surface. Therefore, the HVEM/LIGHT/ BTLA/CD160 signaling axis is expected to present both positive and negative signaling in NKT cells, depending on which receptor/ligand is operated in the context of neighboring interactions. Consequently, HVEM^−/−^ mice exhibit attenuated Con A-induced hepatitis, low serum AST and ALT, and reduced serum IFN-γ^43^. In these mice, α-GalCer-stimulated NKT cells in liver MNCs did not show any differences in surface co-stimulatory or co-inhibitory receptors; however, they did produce higher IL-17 and IL-22 without affecting IFN-γ and TNF-α, promoting tissue repair. Since NKT cells initiate acute hepatitis pathology in Con A-challenged mice, the attenuated phenotype in HVEM^−/−^ mice could be associated with other HVEM-expressing liver MNCs, such as CD4^+^ T cells, in these mice. In this context, accelerated NKT cell activation in CD160^−/−^ mice could be due to increased availability of HVEM on CD4^+^ T cells, which could, in turn, lead to severe inflammation and acute hepatic failure.

Our data based on CD160^−/−^ and mixed bone marrow chimera models highlight that CD160 serves as a co-inhibitory rather than a co-stimulatory receptor on NKT cells. Both WT DC and CD160^−/−^ DC express comparable levels of surface co-stimulatory/co-inhibitory ligands, and exert similar accelerated cytokine production in CD160^−/−^ NKT cells compared with WT NKT cells, confirming the mixed bone marrow chimera results suggesting that the defect is intrinsic to NKT cells, not DCs or surrounding APCs.

Currently, the precise mechanism underlying CD160-mediated negative signals in NKT cells remains unclear. However, CD160 likely either takes over BTLA binding from HVEM^4,44^ or potentiates CD160/BTLA/HVEM binding, thereby dominating co-inhibition of NKT signaling during a slightly later phase of innate immune reactions. Interestingly, CD160^−/−^ NKT cells downregulated surface BTLA during acute hepatitis (Fig. 5b). These data suggest that CD160 may be required for BTLA expression in NKT cells to deliver co-inhibitory signals in normal innate immune responses. Hyperactivation of NKT cells in the absence of CD160 may also be associated with upregulation of CD40L, shifting the balance toward CD40/CD40L-costimulation over HVEM/BTLA co-inhibition (Fig. 5b). In human CD4^+^ T cells, cross-linking of CD160 in the presence of anti-CD3- and anti-CD28-mAb-coated beads suppressed the expression of *IL2, IL6*, and *IL17A* as well as *IL2RA (CD25)* mRNA transcripts without affecting known suppressive genes, *TGFB1, IL10, PDCD1, CD274*, *PDCD1LG2* and *CTLA4*^37^. On preliminary RNAseq analysis, the level of *IL-10* (−4.7-fold) and *CTLA4* (−3.3-fold) was significantly reduced in CD160^−/−^ upon α-GalCer challenge. Since the role of CD160 in NKT cells mimic that in CD4^+^ T cells, similar gene expression changes can be expected in NKT cells and contribute to the co-inhibitory phenotypes of NKT cells (Park et al., unpublished data).

In summary, we have shown that CD160 is an important co-inhibitory receptor in NKT cells during acute innate immune responses. Although its ligand HVEM functions as a bidirectional switch in T cells, whereby the outcome depends on the receptors engaged, the functional consequence of CD160/BTLA/HVEM interactions in NKT cells is co-inhibitory, with deficiency aggravating NKT-mediated acute hepatitis. Therefore, both CD160 and BTLA are required for normal NKT-mediated innate immune responses. Taken together, our findings of CD160 as an additional co-inhibitory receptor in NKT cells shed light on the development of CD160/BTLA/HVEM pathways as potential therapeutic targets for the treatment of acute liver inflammatory diseases.

## Methods

### Mice

Wild type (WT) C57BL/6 mice were purchased from Orient Bio (Orient Bio. Inc., Seongnam, Korea). CD160^−/−^ mice on a C57BL/6 background were kindly provided by Prof. Yang-Xin Fu (UT Southwestern Medical Center, TX, USA). Mice between 8 and 12 weeks of age were used for experiments. All animal experiments were approved by the Institutional Animal Care and Use Committee of Korea University (approval number: KUIACUC-20160518-1) and followed the guidelines and regulations of the Institutional Animal Care and Use Committees of Korea University.

### Cell preparation

The liver was removed from mice after perfusion with DPBS via the hepatic portal vein. Perfused liver homogenates were incubated with collagenase IV (Worthington, NJ, USA) for 15–30 min at 37 °C, and were passed through a 100-μm strainer. Cells were centrifuged at 800× for 5 min and pellets were suspended in 33% Percoll (GE Healthcare Bio-Sciences, PA, USA). The suspension was centrifuged at 800 × *g* for 30 min and RBCs were removed with ACK lysis buffer (Gibco, MA, USA). Lymphocytes from spleens of WT and CD160^−/−^ mice were homogenized using a 70-μm strainer (SPL, Korea) to isolate single cells.

### Analysis of cell activation in vitro

BMDCs were cultured with 10 ng/ml GMCSF (BioLegend, CA, USA) in RPMI1640 with 5% FBS, 100 U/ml penicillin/streptomycin, 0.1% non-essential amino acids, 10 mM HEPES, and 55 μM 2-mercaptoethanol. BMDCs were stimulated with α-GalCer (100 ng/ml) for 16 h and cultured for 48 h with liver MNCs in round-bottom 96-well plates^45^. To measure cytokine production, culture supernatants were analyzed for various cytokine levels using a CBA kit (BD Bioscience) and ELISA Kit (BioLegend). For intracellular staining, liver MNCs were stimulated for 24 h with α-GalCer (100 ng/ml) in the presence of Brefeldin A (BioLegend) or Golgistop (BD Pharmingen, CA, USA).

### Flow cytometry

Fluorochrome-conjugated antibodies against mouse CD3e (clone: 145-2C11, [eB], cat.: #11-0031-85, dilution: 1:300), CD4 (RM4-5, [BL], #100540, 1:800), TCR-β (H57-597, [eB], #11-5961-82, 1:400), NK1.1 (PK136, [eB], #12-5941-83, 1:200; PK136, [eB], #25-5941-81, 1:200), CD160 (ebioCNX46-3, [eB], #12-1601-82, 1:400), CD69 (F23.1, [eB], #11-0691-85, 1:300), CD8a (53-6.7, [eB], #11-0081-82, 1:300), CD19 (1D3, [BD], #551001, 1:500), CD44 (IM7, [BD], #560570, 1:400), CD24 (M1/69, [BL], #101814, 1:1,000), CD28 (37.51, [eB], #25-0281-81, 1:500), CD45 (30-F11, [BL], #103116, 1:800), IL-4 (11B11, [BL], #504117, 1:400), IFN-γ (XMG-1, [BD], #554413, 1:400), BTLA (6F7, [eB], #17-5956-82, 1:400), HVEM (LH1, [eB], #17-5962-82, 1:400), CD178 (MFL3, [eB], #17-5911-82, 1:500), CD80 (16-10A1, [eB], #11-0801-82, 1:400), CD40 (3/23, [BL], #124607, 1:500), CD137 (1AH2, [BD], #558975, 1:200), CD134 (OX-86, [eB], #12-1341-81, 1:400), CD1d (1B1, [BL], #123507, 1:200), CTLA-4 (UC10-4B9, [eB], #12-1522-81, 1:500), PD-1 (J43, [eB], #11-9985-85, 1:300), PD-L1 (10 F.9G2, [BL], #124312, 1:500), CD154 (MR1, [BL], #106510, 1:500), CD95 (clone: 15A7, [eB], #46-0951-80, 1:500), PLZF (9E12, [BL], #145808, 1:500), T-bet (4B10, [eB], #25-5825-80, 1:500), RORγt (B2D, [eB], #17-6981-80, 1:500), CD45.1 (A20, [eB], #45-0453-82, 1:500), CD45.2 (104, [BL], #109822, 1:500), F4/80 (BM8, [eB], #11-4801-82, 1:200), CD11b (M1/70, [eB], #56-0112-82, 1:500), CD11C (N418, [BL], #117310, 1:500), MHCII (M5/114.15.2, [BL], #107630, 1:800), Ly6G (1A8, [BL], #127618, 1:800), and Ly6C (HK1.4, [BL], #128006, 1:500) were purchased from eBioscience [eB], BD Pharmingen [BD], or BioLegend [BL]. PE-labeled CD1d tetramer loaded with α-GalCer was obtained from the NIH tetramer facility. Fixable viability stain 700 (BD Bioscience, CA, USA) or 7AAD (BD Bioscience) was used for dead cell exclusion. Intracellular staining was performed using the Cytofix/Cytoperm kit according to the manufacturer’s protocol. Flow cytometric analysis was performed with FACS Canto II (BD Bioscience) and data were analyzed with FlowJo software (ThreeStar, OR, USA). For measurement of cytokine levels, peripheral blood was collected in vacutainers via retro-orbital bleeding. Levels of cytokines in serum and supernatants were measured with the Mouse Th1/Th2/Th17 Cytokine Kit (BD Biosciences) according to the manufacturer’s instructions. Data were analyzed with the FCAP Array software (Soft Flow, version 3.0).

### Acute hepatitis model

Mice were i.v. or i.p. injected with vehicle or 2 μg α-GalCer (Funakoshi, CA, USA) in 200 μl. Con A (Sigma-Aldrich, MO, USA) was dissolved in PBS and intravenously (i.v.) injected into mice at a sub-lethal concentration (15 mg/kg). For survival studies, a 30 mg/kg lethal dose was applied. For depletion of CD8^+^ T cells, mice were intraperitoneally injected with anti-CD8 mAb (clone: YTS 169.4) 24 h before Con A injection. For blocking of BTLA, mice were intravenously injected with anti-BTLA (clone: pj196, BioXcell, NH, USA) 2 h before α-GalCer (2 μg) injection. For neutralizing of CD1d, mice were intraperitoneally administrated 200 μg of anti-CD1d antibodies (clone: 20H2, BioXcell) on day 1 before α-GalCer (2 μg) injection.

### Generation of mixed bone marrow chimeras

Chimeric mice were generated by whole-body γ-irradiation followed by transfer of bone marrow cells. Briefly, CD45.2 WT mice were irradiated with a dose of 9 Gy and then injected i.v. with 1 × 10^6^ mixed bone marrow cells (ratio 1:1) from the femurs and tibias of CD45.1 WT and CD45.2 CD160^−/−^ 6 h later. Mice were administered antibiotics (neomycin 1.1 mg/ml and polymyxin B sulfate 1000 U/ml in acidic drinking water) for 2 weeks. After 6 weeks, mice were injected i.p. with α-GalCer (2 μg) for 4 h.

### Measurement of aminotransferase activity

Serum alanine aminotransferase (ALT) and aspartate aminotransferase (AST) activity was determined using a commercial kit (BioVision, CA, USA). The absorbance was determined using a microplate spectrophotometer (Spectra Max 190, Molecular Device, CA, USA).

### RNA isolation and quantitative real-time PCR analysis

Total RNA was extracted with TRIzol Reagent (Invitrogen, CA, USA). cDNA was synthesized using a TOPscript™ cDNA Synthesis Kit (Enzynomics, Daejeon, Korea). Real-time PCR was performed with SYBR green (Bio-rad, CA, USA) on a StepOnePlus™ (Applied Biosystems, CA, USA). Gene expression was normalized to the levels of *HPRT* mRNA, and relative expression levels were calculated according to the ΔΔCT method. Genes were amplified using the primers listed in Supplementary Table 1. CD160 primer was purchased from Bio-rad.

### Liver histology

Liver tissue samples were fixed in 10% neutral-buffered formalin (Biosesang Inc, Korea) and embedded in paraffin wax. Tissue sections (7 μm) were stained with hematoxylin and eosin (H&E) or subjected to terminal deoxynucleotidyl transferase dUTP nick end labeling (TUNEL) staining according to the kit manufacturer’s instructions (DeadEnd^TM^, Promega, WI, USA).

### Statistical analyses

All data were presented as the mean ± standard error (S.E.M.). Statistical significance was determined by unpaired two-tailed *t*-tests, or paired *t*-tests. For survival curves, log-rank (Mantel–Cox) test was performed. Significance was defined as *p < 0.05, **p < 0.01, and ***p < 0.001. All analyses were performed using Prism 5.0 software (GraphPad Software, Inc., CA, USA).

### Reporting summary

Further information on research design is available in the Nature Research Reporting Summary linked to this article.

## Supplementary information

Supplementary Information

Reporting Summary

Source Data

## Data Availability

The authors declare that the data supporting the findings of this study are available within the article, supplementary information files and Source Data, or are available upon reasonable requests to the authors.

## References

[1] Anumanthan A (1998). Cloning of BY55, a novel Ig superfamily member expressed on NK cells, CTL, and intestinal intraepithelial lymphocytes. J. Immunol..

[2] Fons P (2006). Soluble HLA-G1 inhibits angiogenesis through an apoptotic pathway and by direct binding to CD160 receptor expressed by endothelial cells. Blood.

[3] Maeda M (2005). Murine CD160, Ig-like receptor on NK cells and NKT cells, recognizes classical and nonclassical MHC class I and regulates NK cell activation. J. Immunol..

[4] Gonzalez LC (2005). A coreceptor interaction between the CD28 and TNF receptor family members B and T lymphocyte attenuator and herpesvirus entry mediator. P Natl Acad. Sci. USA.

[5] Sedy JR (2005). B and T lymphocyte attenuator regulates T cell activation through interaction with herpesvirus entry mediator. Nat. Immunol..

[6] Cheung TC (2009). Unconventional ligand activation of herpesvirus entry mediator signals cell survival (vol 106, pg 6244, 2009). Proc Natl Acad. Sci. USA.

[7] Harrop JA (1998). Antibodies to TR2 (herpesvirus entry mediator), a new member of the TNF receptor superfamily, block T cell proliferation, expression of activation markers, and production of cytokines. J. Immunol..

[8] Tamada K (2000). Modulation of T-cell-mediated immunity in tumor and graft-versus-host disease models through the LIGHT co-stimulatory pathway. Nat. Med..

[9] Blackburn SD (2009). Coregulation of CD8(+) T cell exhaustion by multiple inhibitory receptors during chronic viral infection. Nat. Immunol..

[10] Peretz Yoav, He Zhong, Shi Yu, Yassine-Diab Bader, Goulet Jean-Philippe, Bordi Rebeka, Filali-Mouhim Ali, Loubert Jean-Baptiste, El-Far Mohamed, Dupuy Franck P., Boulassel Mohamed Rachid, Tremblay Cécile, Routy Jean-Pierre, Bernard Nicole, Balderas Robert, Haddad Elias K., Sékaly Rafick-Pierre (2012). CD160 and PD-1 Co-Expression on HIV-Specific CD8 T Cells Defines a Subset with Advanced Dysfunction. PLoS Pathogens.

[11] Viganò Selena, Banga Riddhima, Bellanger Florence, Pellaton Céline, Farina Alex, Comte Denis, Harari Alexandre, Perreau Matthieu (2014). CD160-Associated CD8 T-Cell Functional Impairment Is Independent of PD-1 Expression. PLoS Pathogens.

[12] Le Bouteiller P (2002). Engagement of CD160 receptor by HLA-C is a triggering mechanism used by circulating natural killer (NK) cells to mediate cytotoxicity. Proc. Natl Acad. Sci. USA.

[13] Bendelac A, Schwartz RH (1991). CD4+ and CD8+ T cells acquire specific lymphokine secretion potentials during thymic maturation. Nature.

[14] Godfrey DI, MacDonald HR, Kronenberg M, Smyth MJ, Van Kaer L (2004). NKT cells: what’s in a name?. Nat. Rev. Immunol..

[15] Bendelac A, Savage PB, Teyton L (2007). The biology of NKT cells. Annu Rev. Immunol..

[16] Van Kaer L (2005). α-Galactosylceramide therapy for autoimmune diseases: prospects and obstacles. Nat. Rev. Immunol..

[17] Brossay L (1998). CD1d-mediated recognition of an alpha-galactosylceramide by natural killer T cells is highly conserved through mammalian evolution. J. Exp. Med.

[18] Kawano T (1997). CD1d-restricted and TCR-mediated activation of valpha14 NKT cells by glycosylceramides. Science.

[19] Watarai H, Nakagawa R, Omori-Miyake M, Dashtsoodol N, Taniguchi M (2008). Methods for detection, isolation and culture of mouse and human invariant NKT cells. Nat. Protoc..

[20] Godfrey DI, Stankovic S, Baxter AG (2010). Raising the NKT cell family. Nat. Immunol..

[21] Savage AK (2008). The transcription factor PLZF directs the effector program of the NKT cell lineage. Immunity.

[22] Lee YJ, Holzapfel KL, Zhu J, Jameson SC, Hogquist KA (2013). Steady-state production of IL-4 modulates immunity in mouse strains and is determined by lineage diversity of iNKT cells. Nat. Immunol..

[23] Matsumoto H (2011). Coincidence of autoantibody production with the activation of natural killer T cells in alpha-galactosylceramide-mediated hepatic injury. Immunology.

[24] Paget C (2012). Interleukin-22 Is produced by invariant natural killer T lymphocytes during influenza A virus infection potential role in protection against lung epithelial damages. J. Biol. Chem..

[25] Cerundolo V, Silk JD, Masri SH, Salio M (2009). Harnessing invariant NKT cells in vaccination strategies. Nat. Rev. Immunol..

[26] Iwata A (2010). Protective roles of B and T lymphocyte attenuator in NKT cell-mediated experimental hepatitis. J. Immunol..

[27] Leite-De-Moraes MC (2000). Fas/Fas ligand interactions promote activation-induced cell death of NK T lymphocytes. J. Immunol..

[28] Uldrich AP (2005). NKT cell stimulation with glycolipid antigen in vivo: costimulation-dependent expansion, Bim-dependent contraction, and hyporesponsiveness to further antigenic challenge. J. Immunol..

[29] Vincent MS (2002). CD1-dependent dendritic cell instruction. Nat. Immunol..

[30] Wang JX (2009). Cutting edge: CD28 engagement releases antigen-activated invariant NKT cells from the inhibitory effects of PD-1. J. Immunol..

[31] Zaini J (2007). OX40 ligand expressed by DCs costimulates NKT and CD4+ Th cell antitumor immunity in mice. J. Clin. Investig..

[32] Horney JT, Galambos JT (1977). The liver during and after fulminant hepatitis. Gastroenterology.

[33] Boyer JL (1976). The diagnosis and pathogenesis of clinical variants in viral hepatitis. Am. J. Clin. Pathol..

[34] Kumar V (2013). NKT-cell subsets: Promoters and protectors in inflammatory liver disease. J. Hepatol..

[35] Giustiniani J, Marie-Cardine A, Bensussan A (2007). A soluble form of the MHC class I-specific CD160 receptor is released from human activated NK lymphocytes and inhibits cell-mediated cytotoxicity. J. Immunol..

[36] Tu TC (2015). CD160 is essential for NK-mediated IFN-gamma production. J. Exp. Med.

[37] Cai GF (2008). CD160 inhibits activation of human CD4(+) T cells through interaction with herpesvirus entry mediator (vol 9, pg 176, 2008). Nat. Immunol..

[38] Harrop JA (1998). Herpesvirus entry mediator ligand (HVEM-L), a novel ligand for HVEM/TR2, stimulates proliferation of T cells and inhibits HT29 cell growth. J. Biol. Chem..

[39] Wang Y (2005). The role of herpesvirus entry mediator as a negative regulator of T cell-mediated responses. J. Clin. Investig..

[40] Watanabe N (2003). BTLA is a lymphocyte inhibitory receptor with similarities to CTLA-4 and PD-1. Nat. Immunol..

[41] Miller ML, Sun YL, Fu YX (2009). Cutting edge: B and T lymphocyte attenuator signaling on NKT cells inhibits cytokine release and tissue injury in early immune responses. J. Immunol..

[42] Wang J (2001). The regulation of T cell homeostasis and autoimmunity by T cell-derived LIGHT. J. Clin. Investig..

[43] Wahl C, Wegenka UM, Leithauser F, Schirmbeck R, Reimann J (2009). IL-22-dependent attenuation of T cell-dependent (ConA) hepatitis in herpes virus entry mediator deficiency. J. Immunol..

[44] Sedy JR (2013). CD160 activation by herpesvirus entry mediator augments inflammatory cytokine production and cytolytic function by NK cells. J. Immunol..

[45] Paget C (2007). Activation of invariant NKT cells by toll-like receptor 9-stimulated dendritic cells requires type I interferon and charged glycosphingolipids. Immunity.

