# Supplementary material for: CD160 serves as a negative regulator of NKT cells in acute hepatic injury
Source: Nat Commun. 2019 Jul 22;10:3258. doi: 10.1038/s41467-019-10320-y (PMC6646315; doi:10.1038/s41467-019-10320-y)
Supplement: Supplementary file 1 — Supplementary Information [file 41467_2019_10320_MOESM1_ESM.pdf]

CD160 serves as a negative regulator of NKT cells in acute hepatic injury

Kim et al.

Supplementary Information

Supplementary Figures

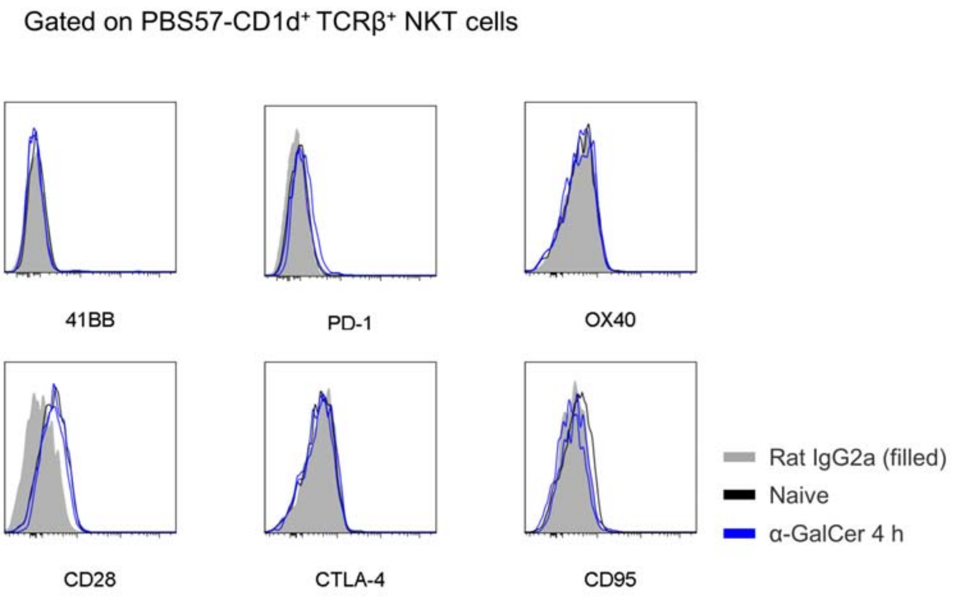

Supplementary Figure 1. Flow cytometric analysis of surface co-stimulatory and co-inhibitory receptors on NKT cells after α-GalCer-injection

The surface co-stimulatory and co-inhibitory receptors on liver NKT cells (TCRβ^{+} PBS57-CD1d^{+}) were analyzed by flow cytometry for 4 hour before and after α-GalCer injection (2μ g).

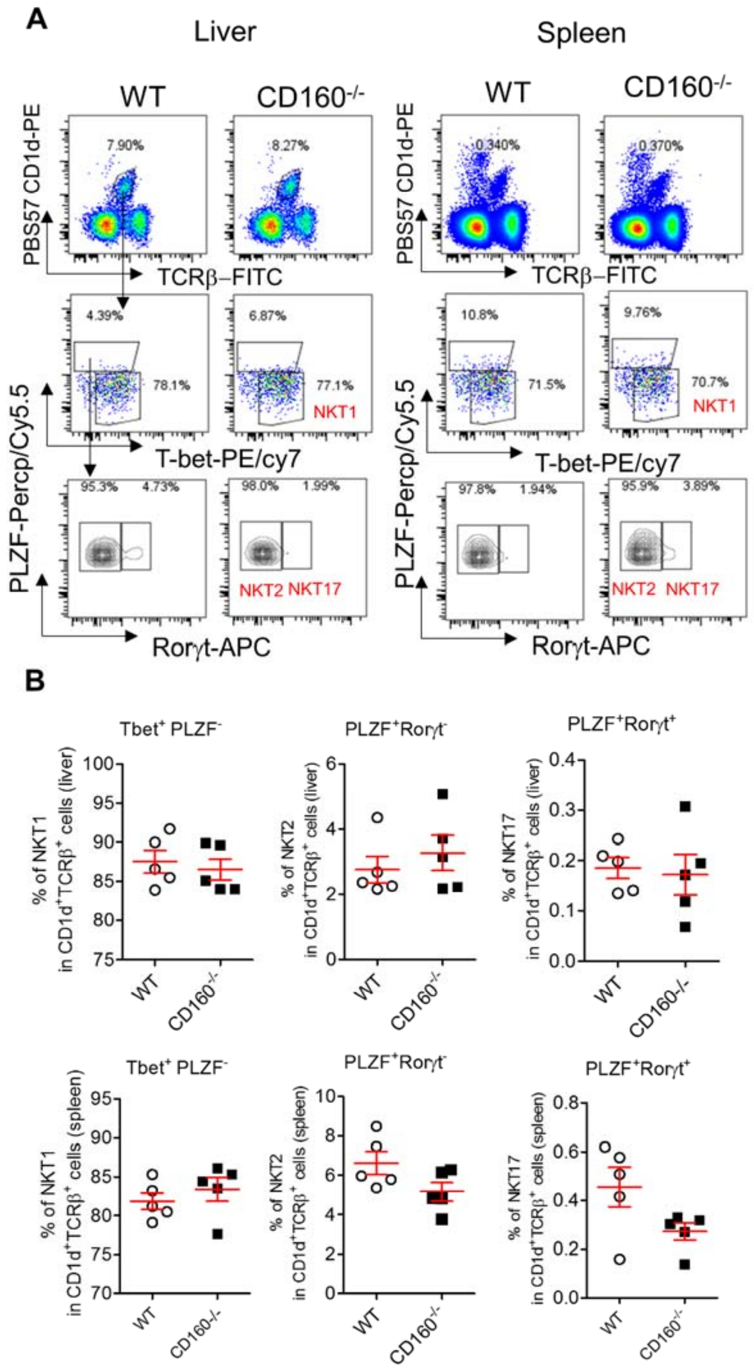

Supplementary Figure 2. FACS analysis of NKT1, NKT2, and NKT17 cells in the liver and spleen from WT and CD160$^{-/-}$ mice.

(A) NKT cells were gated on PLZF, T-bet and RORγt and the frequency of NKT1, NKT2, and NKT17 cells was measured. **(B)** Graphs show the frequency of NKT1, NKT2, and NKT17 cells between WT and CD160$^{-/-}$ mice (n=5).

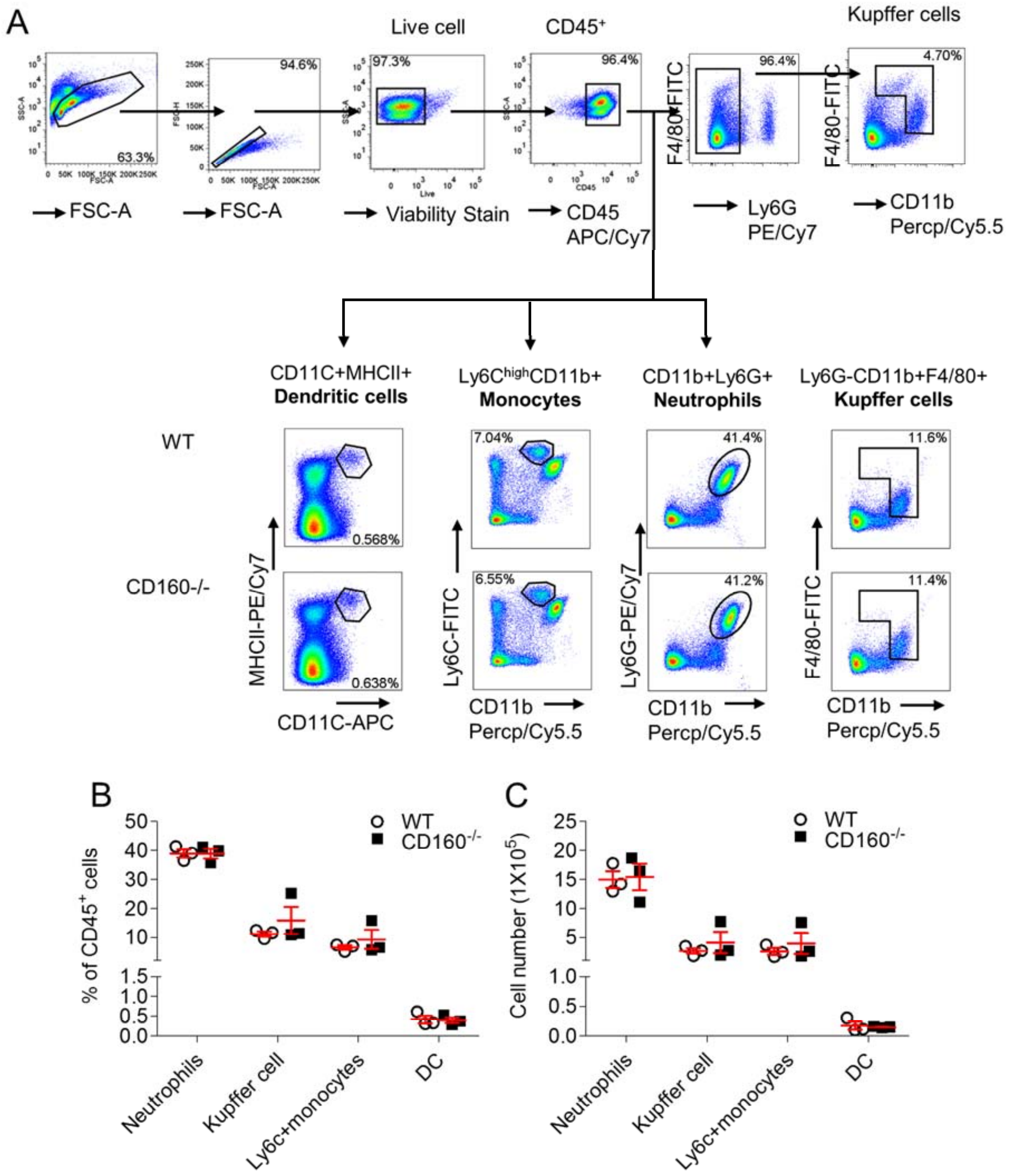

Supplementary Figure 3. Strategy for defining myeloid cells in the liver.

(A) Flow cytometry plots show the gating strategy for identification of Kupffer cells, Ly6C^{high} monocytes, dendritic cells, and neutrophils from liver MNCs. FSC, forward scatter; SSC, side scatter. Ly6G^{+}CD11b^{+} neutrophils, Ly6G^{-}F4/80^{+}CD11b^{+} Kupffer cells, CD11b^{+}Ly6C^{high} monocytes and CD11C^{+}MHCII^{+} dendritic cells infiltrated into the liver 4 hours following i.p. injection of α-GalCer (2 μg) were shown on flow cytometry. Graph showing the frequency **(B)** and absolute cell numbers **(C)** of each hepatic cell population (neutrophils, Kupffer cells, monocytes, and dendritic cells) isolated from WT and CD160$^{-/-}$ mice (n=3).

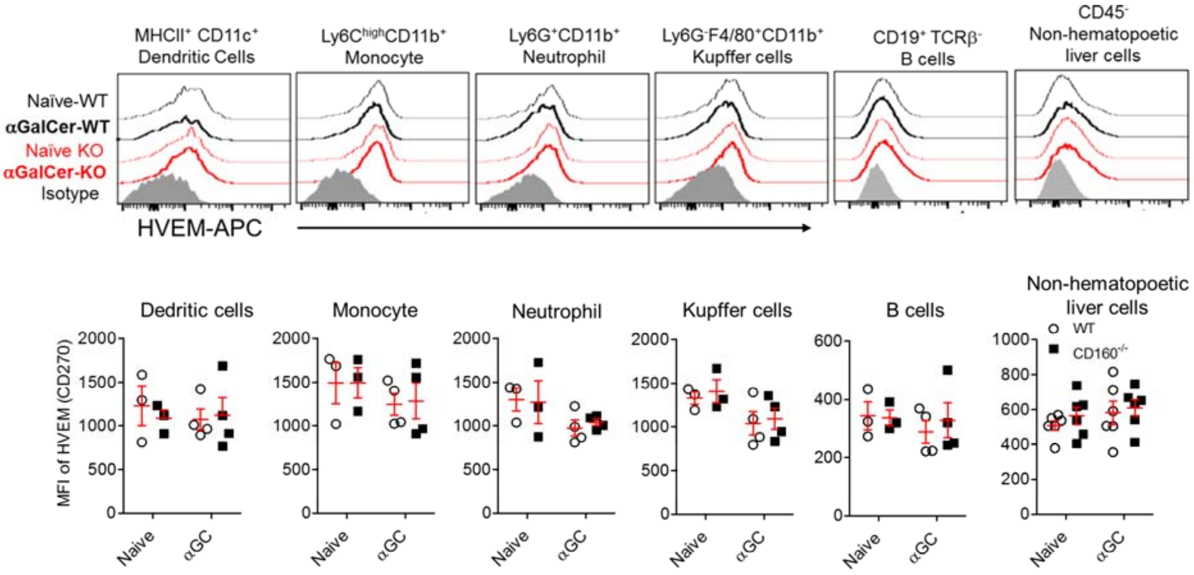

Supplementary Figure 4. HVEM expression on liver MNCs.

(A) Representative flow cytometric plots showing the surface expression of HVEM on various liver MNCs isolated from WT and CD160$^{-/-}$ mice at 4 hours before and after 2 µg of α-GalCer administration in vivo. **(B)** MFI of surface HVEM was calculated and plotted as graphs (mean ± S.E.M., n=3-6 per group).

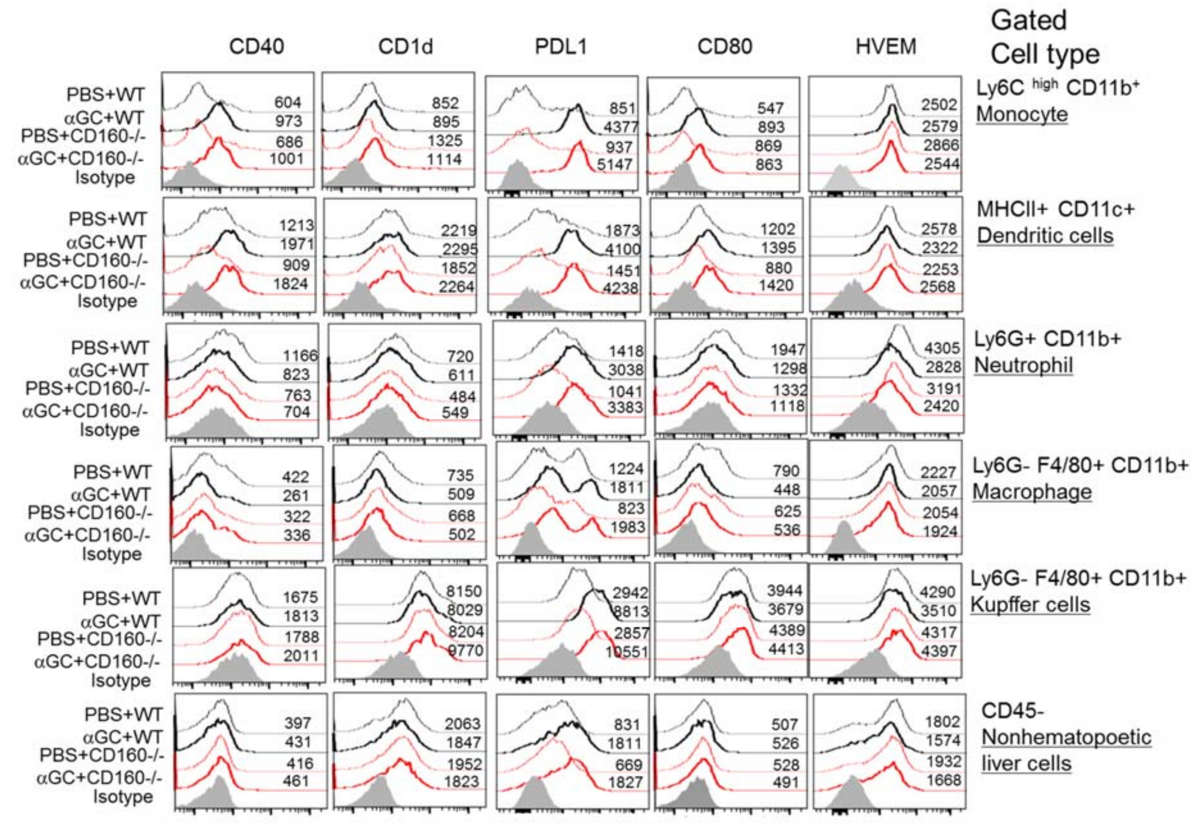

Supplementary Figure 5. Flow cytometric analysis of co-stimulatory and co-inhibitory receptor expression in liver cells from WT and CD160$^{-/-}$ mice.

Surface expression of co-stimulatory and co-inhibitory receptors on various subsets of liver MNCs from WT and CD160$^{-/-}$ mice 4 hours before and after 2-μg α-GalCer challenge in vivo. Representative FACs plots from three independent experiments are shown.

CD160-/- mice sensitive to Con A-induced hepatitis in absence of CD8+ T cells

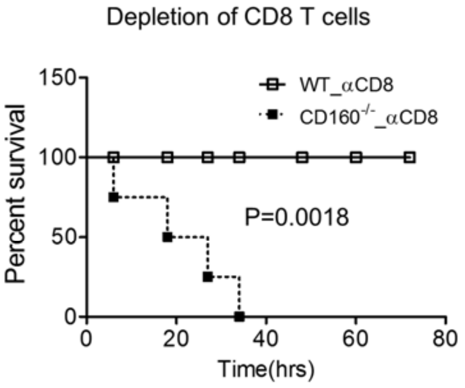

Supplementary Figure 6. CD160$^{-/-}$ mice are sensitive to Con A in the absence of CD8^{+} T cells.

CD8^{+} T cells were depleted with anti-CD8 mAb (YTS 169.4) 24 hours before Con A injection. CD8^{+} T cell-depleted WT and CD160$^{-/-}$ mice were intravenously injected with 30 mg/kg Con A and survival rates were recorded (n=7 per group). *P<0.05, **P<0.01 with the Log-rank (Mantel-Cox) Test.

Supplementary Table 1. Real-time PCR primers for gene expression analysis

Gene	Orientation	Primer Sequence (5'-3')
HPRT	Forward	CACAGGACTAGAACACCTGC
	Reverse	GCTGGTGAAAAGGACCTCT
IFN-γ	Forward	GGCCATCAGCAACAACATAAGCGT
	Reverse	TGGGTTGTTGACCTCAAACTTGGC
IL-6	Forward	GAGGATACCACTCCCAACAGACC
	Reverse	AAGTGCATCATCGTTGTTCATACA
IL-4	Forward	AGATGGATGTGCCAAACGTCCTCA
	Reverse	AATATGCGAAGCACCTTGGAAGCC
TNF	Forward	ATGAGAAGTTCCCAAATGGC
	Reverse	CTCCACTTGGTGGTTTGCTA
