# Supplementary material for: CD160 serves as a negative regulator of NKT cells in acute hepatic injury
Source: Nat Commun. 2019 Jul 22;10:3258. doi: 10.1038/s41467-019-10320-y (PMC6646315; doi:10.1038/s41467-019-10320-y)
Supplement: Supplementary file 2 — Reporting Summary [file 41467_2019_10320_MOESM2_ESM.pdf]

nature research

Corresponding author(s): Kyung-Mi Lee

Last updated by author(s): 03-15-2019

Reporting Summary

Nature Research wishes to improve the reproducibility of the work that we publish. This form provides structure for consistency and transparency in reporting. For further information on Nature Research policies, see Authors & Referees and the Editorial Policy Checklist.

Statistics

For all statistical analyses, confirm that the following items are present in the figure legend, table legend, main text, or Methods section.

n/a	Confirmed	
☐	☒	The exact sample size (n) for each experimental group/condition, given as a discrete number and unit of measurement
☐	☒	A statement on whether measurements were taken from distinct samples or whether the same sample was measured repeatedly
☐	☒	The statistical test(s) used AND whether they are one- or two-sided<br>*Only common tests should be described solely by name; describe more complex techniques in the Methods section.*
☐	☒	A description of all covariates tested
☐	☒	A description of any assumptions or corrections, such as tests of normality and adjustment for multiple comparisons
☐	☒	A full description of the statistical parameters including central tendency (e.g. means) or other basic estimates (e.g. regression coefficient) AND variation (e.g. standard deviation) or associated estimates of uncertainty (e.g. confidence intervals)
☐	☒	For null hypothesis testing, the test statistic (e.g. F, t, r) with confidence intervals, effect sizes, degrees of freedom and P value noted<br>*Give P values as exact values whenever suitable.*
☒	☐	For Bayesian analysis, information on the choice of priors and Markov chain Monte Carlo settings
☒	☐	For hierarchical and complex designs, identification of the appropriate level for tests and full reporting of outcomes
☒	☐	Estimates of effect sizes (e.g. Cohen's d, Pearson's r), indicating how they were calculated

Our web collection on statistics for biologists contains articles on many of the points above.

Software and code

Policy information about availability of computer code

Data collection	Flow cytometric analysis was performed with FACS Diva software (BD Bioscience).
Data analysis	Flow cytometric data were analyzed with FlowJo software (ThreeStar, USA).<br>Cytokine bead array (CBA) data were analyzed with the FCAP Array software (Soft Flow, version 3.0).<br>Statistical analyses were performed using Prism software (GraphPad Prism 5.0).

For manuscripts utilizing custom algorithms or software that are central to the research but not yet described in published literature, software must be made available to editors/reviewers. We strongly encourage code deposition in a community repository (e.g. GitHub). See the Nature Research guidelines for submitting code & software for further information.

Data

Policy information about availability of data

All manuscripts must include a data availability statement. This statement should provide the following information, where applicable:

- Accession codes, unique identifiers, or web links for publicly available datasets
- A list of figures that have associated raw data
- A description of any restrictions on data availability

The data that support the findings of this study are available from the corresponding author upon reasonable request.

Field-specific reporting

Please select the one below that is the best fit for your research. If you are not sure, read the appropriate sections before making your selection.

☒ Life sciences ☐ Behavioural & social sciences ☐ Ecological, evolutionary & environmental sciences

For a reference copy of the document with all sections, see nature.com/documents/nr-reporting-summary-flat.pdf

Life sciences study design

All studies must disclose on these points even when the disclosure is negative.

Sample size	All experimental sample sizes were determined according to the previous published reports.
Data exclusions	No data were excluded from the analysis.
Replication	All experimental replications are included in the figure legend.
Randomization	Allocation into the experimental group was not random because the experiments were performed in age and gender matched and gene matched mice.
Blinding	Investigators were blinded to group allocation during experiment and data analysis

Reporting for specific materials, systems and methods

We require information from authors about some types of materials, experimental systems and methods used in many studies. Here, indicate whether each material, system or method listed is relevant to your study. If you are not sure if a list item applies to your research, read the appropriate section before selecting a response.

Materials & experimental systems

n/a	Involved in the study	
☐	☒	Antibodies
☒	☐	Eukaryotic cell lines
☒	☐	Palaeontology
☐	☒	Animals and other organisms
☒	☐	Human research participants
☒	☐	Clinical data

Methods

n/a	Involved in the study	
☒	☐	ChIP-seq
☐	☒	Flow cytometry
☒	☐	MRI-based neuroimaging

Antibodies

Antibodies used	For Flow cytometric analysis:<br>Fluorochrome-conjugated antibodies against mouse CD3e (clone: 145-2C11, (eB), cat. #11-0031-85, dilution: 1:300), CD4 (RM4-5, (BL), #100540, 1:800), TCR-β (H57-597, (eB), #11-5961-82, 1:400), NK1.1 (PK136, (eB) #12-5941-83, 1:200: PK136, (eB), #25-5941-81, 1:200), CD160 (ebioCNX46-3, (eB), #12-1601-82, 1:400), CD69 (F23.1, (eB), #11-0691-85, 1:300), CD8a (53–6.7, (eB), #11-0081-82, 1:300), CD19 (1D3, (BD), #551001, 1:500), CD44 (IM7, (BD), #560570, 1:400), CD24 (M1/69, (BL), #101814, 1:1000), CD28 (37.51, (eB), #25-0281-81, 1:500), CD45 (30-F11, (BL), #103116, 1:800), IL-4 (11B11, (BL), #504117, 1:400), IFN-γ (XMG-1, (BD), #554413, 1:400), BTLA (6F7, (eB), #17-5956-82, 1:400), HVEM (LH1, (eB), #17-5962-82, 1:400), CD178 (MFL3, (eB), #17-5911-82, 1:500), CD80 (16-10A1, (eB), #11-0801-82, 1:400), CD40 (3/23, (BL), #124607, 1:500), CD137 (1AH2, (BD), #558975, 1:200), CD134 (OX-86, (eB), #12-1341-81), CD1d (1B1, (BL), #123507, 1:200), CTLA-4 (UC10-4B9, (eB), #12-1522-81, 1:500), PD-1 (J43, (eB), #11-9985-85, 1:300), PD-L1 (10F.9G2, (BL), #124312, 1:500), CD154 (MR1, (BL), #106510, 1:500), CD95 (clone: 15A7, (BL), #46-0951-80, 1:500), PLZF (9E12, (BL), #145808,1:500), T-bet (4B10, (eB), #25-5825-80, 1:500), Ror?t (B2D, (eB), #17-6981-80, 1:500), CD45.1 (A20, (eB), #45-0453-82, 1:500), CD45.2 (104, (BL), #109822, 1:500), F4/80 (BM8, (eB), #11-4801-82, 1:200), CD11b (M1/70, (eB), #56-0112-82, 1:500), CD11C (N418, (BL), #117310, 1:500), MHCII (M5/114.15.2, (BL), #107630, 1:800), Ly6G (1A8, (BL), #127618, 1:800), and Ly6C (HK1.4, (BL), #128006, 1:500) were purchased from eBioscience (eB), BD Pharmingen (BD), or BioLegend (BL). The PE-labeled CD1d tetramer (1:300) loaded with α-GalCer was obtained from the NIH tetramer facility.<br><br>For depletion or blocking (in vivo)<br>anti-BTLA (clone: pj196, BioXcell), anti-CD1d antibodies (clone: 20H2, BioXcell)
Validation	All antibodies were validated by manufacturer's recommendation.

Animals and other organisms

Policy information about studies involving animals; ARRIVE guidelines recommended for reporting animal research

Laboratory animals	C57BL/6 mice were purchased from Orient Bio (Orient Bio. Inc., Seongnam, Korea). CD160-/- mice on a C57BL/6 background were kindly provided by Prof. Yang-Xin Fu (UT Southwestern Medical Center, USA).
Wild animals	This study did not include wild animals.
Field-collected samples	This study did not include field-collected samples.
Ethics oversight	All animal experiments have been approved by the Institutional Animal Care and Use Committee of Korea University (Approval number: KUIACUC-20160518-1) and following the guideline and regulations of the Institutional Animal Care and Use Committees of Korea University.

Note that full information on the approval of the study protocol must also be provided in the manuscript.

Flow Cytometry

Plots

Confirm that:

☒ The axis labels state the marker and fluorochrome used (e.g. CD4-FITC).

☐ The axis scales are clearly visible. Include numbers along axes only for bottom left plot of group (a 'group' is an analysis of identical markers).

☒ All plots are contour plots with outliers or pseudocolor plots.

☒ A numerical value for number of cells or percentage (with statistics) is provided.

Methodology

Sample preparation	Perfused liver homogenates were incubated with collagenase IV (Worthington) for 15-30 min at 37°C, and were passed through a 100-µm strainer. Cells were centrifuged at 800 g for 5 min and the pellets were suspended in 33% Percoll (GE Healthcare Bio-Sciences, PA, USA). The suspension was centrifuged at 800 g for 30 min and RBCs were removed with ACK lysis buffer (Gibco, MA, USA).<br>Lymphocytes from spleens were homogenized using 70µm strainer (SPL, Korea) to isolate single cells.
Instrument	All flow cytometric data were aquired with BD Canto II cytometer.
Software	All flow cytometric data were analyzed using Flow Jo software (Flow Jo v10).
Cell population abundance	We did not use cell sorting method in this paper.
Gating strategy	Flow cytometry gating strategy to identify the various cell type from thymus, liver and spleen. Live mononuclear cells were gating using SSC-A and FSC-A channel and fixable viability stain solution (BD horizon). And then doublets were excluded by FSC-A and FSC-H gating. Hematopoetic cells were gating using CD45 staining. Gating strategies are shown in Figure2 and supplement figure 3

☒ Tick this box to confirm that a figure exemplifying the gating strategy is provided in the Supplementary Information.
